# Discrimination of multilocus sequence typing-based *Campylobacter jejuni* subgroups by MALDI-TOF mass spectrometry

**DOI:** 10.1186/1471-2180-13-247

**Published:** 2013-11-07

**Authors:** Andreas Erich Zautner, Wycliffe Omurwa Masanta, Abdul Malik Tareen, Michael Weig, Raimond Lugert, Uwe Groß, Oliver Bader

**Affiliations:** 1 UMG-Labor/Institut für Medizinische Mikrobiologie, Universitätsmedizin Göttingen, Kreuzbergring 57, 37075, Göttingen, Germany; 2 UMG-Labor/Institut für Klinische Chemie - Zentrallabor, Universitätsmedizin Göttingen, Robert-Koch-Straße 40, 37075, Göttingen, Germany

**Keywords:** MALDI-TOF species identification, Phyloproteomics, Multilocus sequence typing, MLST, Intact cell mass spectrometry, ICMS, Principal component analysis, PCA, *Campylobacter jejuni*

## Abstract

**Background:**

*Campylobacter jejuni*, the most common bacterial pathogen causing gastroenteritis, shows a wide genetic diversity. Previously, we demonstrated by the combination of multi locus sequence typing (MLST)-based UPGMA-clustering and analysis of 16 genetic markers that twelve different *C. jejuni* subgroups can be distinguished. Among these are two prominent subgroups. The first subgroup contains the majority of hyperinvasive strains and is characterized by a dimeric form of the chemotaxis-receptor Tlp7_m+c_. The second has an extended amino acid metabolism and is characterized by the presence of a periplasmic asparaginase (*ansB*) and gamma-glutamyl-transpeptidase (*ggt*).

**Results:**

Phyloproteomic principal component analysis (PCA) hierarchical clustering of MALDI-TOF based intact cell mass spectrometry (ICMS) spectra was able to group particular *C. jejuni* subgroups of phylogenetic related isolates in distinct clusters. Especially the aforementioned Tlp7_m+c_^+^ and ansB^+^/ *ggt*^*+*^ subgroups could be discriminated by PCA. Overlay of ICMS spectra of all isolates led to the identification of characteristic biomarker ions for these specific *C. jejuni* subgroups. Thus, mass peak shifts can be used to identify the *C. jejuni* subgroup with an extended amino acid metabolism.

**Conclusions:**

Although the PCA hierarchical clustering of ICMS-spectra groups the tested isolates into a different order as compared to MLST-based UPGMA-clustering, the isolates of the indicator-groups form predominantly coherent clusters. These clusters reflect phenotypic aspects better than phylogenetic clustering, indicating that the genes corresponding to the biomarker ions are phylogenetically coupled to the tested marker genes. Thus, PCA clustering could be an additional tool for analyzing the relatedness of bacterial isolates.

## Background

The Gram-negative bacterium *Campylobacter jejuni*, belonging to the class of *Epsilon Proteobacteria*, is the leading cause for bacterial gastroenteritis and Guillain-Barré-syndrome (GBS) worldwide [1].

Over the years, it has become apparent that different subtypes of *C. jejuni* are associated with different manifestations of disease. Therefore, several *Campylobacter*-subtyping methods have been established. The first, and for a long time the gold standard, was serotyping by slide agglutination using heat-stable and heat-labile antigens [2-5]. Using this methodology, the Lior serotype 4 was found to be associated with acute campylobacteriosis in the majority of cases in Germany, whereas GBS was most strongly associated with Lior serotype 11 [6]. Later phagetyping schemes [7] and restriction fragment length polymorphisms like amplified fragment length polymorphism fingerprinting (AFPL) [8], ribotyping [9], as well as pulsed field gel electrophoresis [10] were used for epidemiological typing.

Today these methods play a minor role in studying *Campylobacter* epidemiology. Instead, sequence-based methods, such as multi locus sequence typing (MLST) [11] and the sequencing of the short variable region of the flagellin A gene (*flaA*-SVR sequencing) [12] are widely used.

Among *C. jejuni* isolates of human origin the most frequent clonal complexes (CC) are CC 21 and CC 45 [13,14]. These two prominent isolate groups differ significantly from each other in various aspects. For one, differences in the stress responses of these two MLST-CC groups were observed. Isolates of CC 21 were more tolerant to extreme temperatures as compared to CC 45 isolates [15] while CC 45 isolates showed increased survival in oxidative and freeze stress models [15]. These differences in stress responses may be the reason for the establishment of certain *C. jejuni* subgroups in defined hosts, environments, and thus the spread over different transmission routes. The finding that acute *Campylobacter*-diarrhea cases caused by CC 21 or CC 45 isolates show different temporal distributions supports this hypothesis [14]. While *C. jejuni* isolates of CC 45 are more prevalent during the early summer months obviously following an environmental transmission route, campylobacteriosis caused by CC 21 isolates are reported more or less consistently throughout the whole year, with a peak during late summer months [16] and with a clear association to infected cattle [17]. The combination of MLST with isolate-profiling for sixteen genetic markers: *ansB*, *dmsA*, *ggt*, *cj1585c*, *cjj81176-1367/71* (*cj1365c*), *tlp7*_*m+c*_ (*cj0951c* plus *cj0952c*), *cj1321-cj1326*, *fucP*, *cj0178*, *cj0755/cfrA*, *ceuE*, *pldA*, *cstII*, and *cstIII* lead to a more detailed subgrouping of the *C. jejuni* population discriminating twelve *C. jejuni* subgroups [18,19].

Recently, matrix-assisted laser desorption ionization-time of flight mass spectrometry (MALDI-TOF MS)-based intact cell mass spectrometry (ICMS) has advanced to be a widely used routine species identification tool for cultured bacteria and fungi [20-22]. This technique also allows the accurate identification of *Campylobacter* and *Arcobacter* species [23].

Moreover, MALDI-TOF MS also has the potential to characterize strains at the subspecies level [24], and hence could act as a useful tool for taxonomy and epidemiology [25]. For example, we were recently able to demonstrate that it is possible to separate typhoid from non-typhoid *Salmonella enterica* subspecies *enteria* serotypes [26].

To investigate the potential of ICMS to discriminate between different *C. jejuni* isolate subgroups with differences in host adaptation and pathogenic potential, we used well-characterized *C. jejuni* isolates [18,19] representing different phylogenetic groups. Especially the discrimination of these isolates positive for the periplasmic gamma-glutamyl-transpeptidase (*ggt)* but negative for the fucose permease (*fucP*) associated with a higher rate of hospitalizations and bloody diarrhea [27] stood in the focus of this approach as compared to MLST and the estimated marker gene profiles in this study.

## Results

### Classification results

A total of 104 *C. jejuni* previously characterized and MLST-typed isolates of either human, bovine, chicken or turkey origin were re-identified using standard procedure ICMS. All isolates were identified as *C. jejuni* with MALDI Biotyper score values ≥2.000.

### PCA analysis of *Campylobacter jejuni* isolates

**Figure 1:**
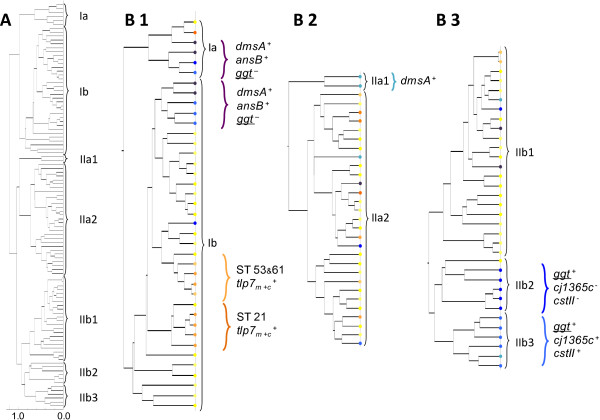
**Dendrogram based on relationships obtained from PCA analysis of the ICMS spectra. (A)** Global cluster analysis of *C. jejuni* isolates. **B1-3**: Enlargement of major clusters, the overall majority of isolates is positive for the marker genes *cj1365c*, *cj1585c*, *cj1321-6*, *fucP*, *cj0178*, and *cj0755* positive but *dmsA*-, *ansB*- and *ggt*-negative (different shades of yellow); **B1**: one cluster of *dmsA*^+^, *ansB*^+^ but *ggt*^-^*C. jejuni* isolates in subtree Ia and a second cluster of dmsA^+^, ansB^+^ but ggt^-^*C. jejuni* isolates in subtree Ib (blue & violet); cluster of CC 53 & CC 61 isolates with the dimeric form of the formic acid specific chemotaxis receptor Tlp7_m+c_ (beige); cluster of Tlp7_m+c_^+^ CC 21 isolates – all of bovine origin (orange); **B2**: small cluster of *dmsA*^+^ and *cstII*^+^ isolates belonging to MLST-CC 1034 (teal) **B3**: The cluster of *ggt*^+^ isolates splits in two subclusters, which differ in *cj1365c* and *cstII* (dark and light blue). The relatedness of *C. jejuni* isolates in the ICMS spectra-based PCA-tree reflects the isolates subgroup affiliation & MLST CC/ST.

In order to determine whether the *C. jejuni* isolate groups as defined by similar marker gene profiles could also be discriminated by their ICMS-spectra, the spectra obtained were clustered by PCA and their phyloproteomic relatedness analyzed. In all four biologically independent analyses we obtained comparable phylogenetic distances of the different isolates by PCA considering the existing degrees of freedom at particular dendrogram nodes (Figure 1).

With only four singular outliners, isolates positive for *dmsA* and *ansB* formed distinct groups within the subclusters Ia, Ib1, and IIb (Figure 1). The corresponding marker gene profiles revealed that nearly all *dmsA* and *ansB* positive isolates in subclusters Ia and Ib1 were *ggt*-negative, whereas nearly all *ggt*-positive isolates formed a combined subcluster IIb2 + IIb3 (Additional file 1: Table S1). Isolates in cluster IIb2 were typically *cstII* and *cj1365c* negative, whereas IIb3 isolates were typically positive for these two genetic markers.

The vast majority of the isolates, predominantly positive for the marker genes *cj1365c*, *cj1585c*, *cj1321-6*, *fucP*, *cj0178* and *cj0755*, were distributed across the clusters, however a subset of isolates expressing the dimeric variant of the TLP7-receptor TLP7_m+c_ formed two distinct sets in the neighboring subclusters Ib2 (ST 53 & 61 isolates) and Ib3 (ST 21 isolates).

**Figure 2:**
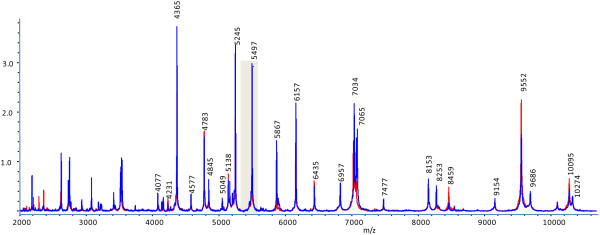
**Overlay of ICMS spectra (Overview of entire MALDI-TOF MS spectrum).** General overview of the whole MALDI-TOF-MS spectrum of the *C. jejuni* strains NCTC 11168 (red) and 81-176 (blue). The numbers above the peaks indicate their m/z-value. The shaded area marks the mass range that is detailed in Figure 3.

**Figure 3:**
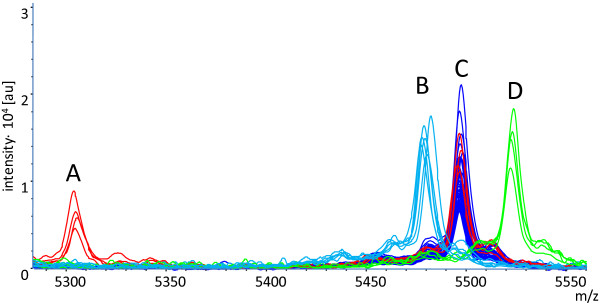
**Overlay of ICMS spectra (Detail of Figure**2**).** Overlay of ICMS spectra of all isolates led to the identification of characteristic peaks for specific *C. jejuni* subgroups. Peak **A** (m/z = 5303; red) is specific for isolates of MLST-ST 21 expressing a dimeric form of the formic acid specific chemotaxis receptor Tlp7_m+c_. The majority of isolates shows a peak at m/z = 5496 (peak **C**, dark blue). *Ggt*- and *cj1365c*-postive isolates (MLST-ST 21) show a shift of this peak to m/z = 5479 (peak **B**, light blue), whereas *ggt*-positive but *cj1365c*- and *cstII*-negative isolates (MLST-ST 45) show a shift of this peak to m/z = 5523 (peak **D**, green).

In an overlay of the spectra from all isolates included in this study (Figure 2) one particular mass (A, m/z = 5303) separated CC 21/ST 21 *C. jejuni* isolates positive for TLP7_m+c_ and of bovine origin from all others (Figure 3). Two additional masses separated *ggt*-positive *C. jejuni* isolates from *ggt*-negative ones. The majority of isolates displayed a peak at m/z = 5496 (C), which is replaced by neighboring peaks in specific isolates. The *ggt*- and *cj1365c*-postive *C. jejuni* isolates (MLST-ST 22) showed a shift of this peak from m/z = 5496 to ~5479 (B). In contrast to that the *ggt*-positive but *cj1365c*- and *cstII*-negative isolates (MLST ST-45) showed a shift of this peak into the opposite direction to m/z = 5523 (D).

### Comparison of phylogenetic and phyloproteomic analyses

**Figure 4:**
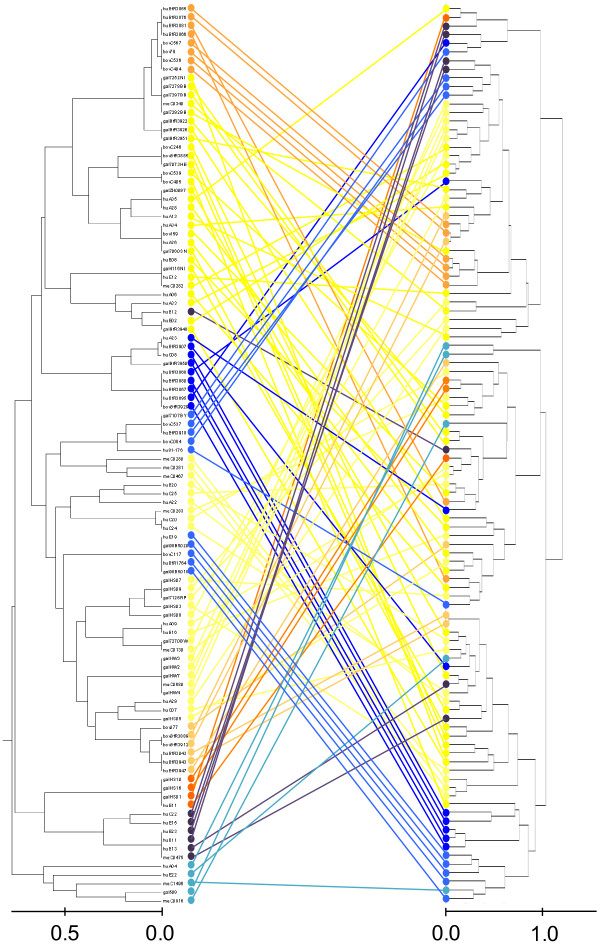
**Comparison of the ICMS-spectra-based PCA-phyloproteomic tree with the phylogenetic MLST-based UPGMA-tree.** Most of the Tlp7_m+c_^+^ isolates cluster together in the ICMS-spectra-based PCA-dendrogram as well as the MLST-based UPGMA-tree (orange); ggt^+^ isolates of MLST-CC 22, CC 45, and CC-283 form a common cluster in the PCA-tree (IIb2 + 3) whereas MLST-CC 42 isolates (mixed ggt^+/-^) cluster together with MLST-CC 257 isolates (*dmsA*^+^, *ansB*^+^ but *ggt*^-^).

To determine if there was a more global correlation between phyloproteomic and phylogenetic relatedness, the two dendrograms obtained by PCA and MLST clustering were compared (Figure 4).

The MLST-based UPGMA-dendrogram splits at two bifurcations into a minor and a major group. At the third bifurcation the remaining isolates form two approximately equal groups. In each of both groups, subgroups positive for *dmsA* and *ansB* and predominantly also for *ggt* are present.

In the ICMS-spectra-based PCA-dendrogram the *ggt*-positive isolates of both subgroups form a common cluster combined of two subgroup specific subclusters, whereas most of the *ggt*-negative isolates form a separate cluster together with the *dmsA*- and *ansB*-positive but *ggt*-negative isolates of that cluster, which branched off at the second bifurcation of the MLST-based UPGMA-dendrogram (MLST-CC 257).

The vast majority of the *C. jejuni* isolates of both groups formed by MLST-CC 21, 48, 49, 206, and 446 as well as MLST-CC 52, 353, 354, 443, 658, and 61 is positive for the marker genes *cj1365c*, *cj1585c*, *cj1321-6*, *fucP*, *cj0178* and *cj0755*. These isolates, with comparable marker gene profile, mix in the ICMS-spectra-based PCA-dendrogram despite of their phylogenetic distance, as noted above. One obvious exception is a group of MLST-ST 21 isolates of bovine origin expressing TLP7_m+c_, which forms a common subcluster in the PCA-subcluster Ib. Finally, there is very small cluster with a significant phylopreteomic distance (IIa1) of *dmsA*^+^ and *cstII*^+^ isolates belonging to MLST-CC 1034.

## Discussion

Today, phylogenetic methods like MLST [11] and *flaA*-SVR sequencing [12] are considered to be the standard typing methods for *C. jejuni* isolates. Thus, every new classification technique must be compared with those genomic classifications [25]. However, the genomic methods reflect some phenotypic aspects only insufficiently.

In this context, MALDI-TOF MS-based ICMS has recently advanced to be a widely used routine species identification tool for cultured bacteria and fungi [20-22]. In contrast to species identification by ICMS, subtyping within a single species (or differentiation between extremely close related species) is a more subtle process. Nevertheless, several examples already do exist proving the applicability of this method for isolate differentiation at the subspecies level, for example it was shown that methicillin-resistant and methicillin-susceptible *Staphylococcus aureus* strains can be discriminated by ICMS [28]. ICMS can also be used to differentiate between the Lancefield groups A, B, C, and G of *Streptococci*[29,30]. Other examples are the subtyping of *Listeria monocytogenes*[31], *Salmonella enterica*[26,32,33], *Yersinia enterocolitica*[34], and *Stenotrophomonas* spp. [35].

The discrimination between the different *Campylobacter* and closely related species is well established and species-specific mass spectra are integrated in routine databases [23,36-39]. It has also been demonstrated that shifts in biomarker masses, which are observable in MALDI-TOF spectra due to amino acid substitutions caused by nonsynonomous mutations in the biomarker gene, can be used to discriminate between the *C. jejuni* subspecies *C. jejuni subsp. jejuni* and *C. jejuni subsp. doylei*[37,40].

As noted above the *C. jejuni* population is divided into two major isolate groups, which differ significantly from each other in stress response, transmission route, host tropism, temporal distribution, and pathogenic potential for humans. These two (including related *C. jejuni* subgroups) are associated with specific genetic markers. CC 21 isolates as well as the vast majority of other *C. jejuni* isolates are positive for *cj1365c* (*cjj81176-1367/1371*), *cj1585c*, *cj1321-cj1326*, *fucP*, *cj0178*, and *cj0755*/*cfrA* (Additional file 2: Table S2) [18,19].

In contrast to that, MLST-CC 45 isolates and the related isolates of the MLST-CC 22, 42, and 283 are predominantly negative for these marker genes; with the exception that MLST-CC 22 and 42 isolates harbor *cj1365c*. In these isolates the oxidoreductase gene *cj1585c* is replaced by the tripartite anaerobic dimethyl sulfoxide oxidoreductase *dmsA to –D* facilitating an alternative anaerobic metabolic pathway. Additionally this isolate group has an extended amino acid metabolism and is characterized by the presence of *ggt* and *ansB*. The *cj1365c*-positive isolates of MLST-CC 22 and 42 are also *cstII*-positive, whereas MLST-CC 45 and 282 isolates have no LOS-sialyltransferase genes [18,19]. Theses isolates positive for *ggt* but negative for *fucP* could be significantly associated with a higher rate of hospitalizations and bloody diarrhea and bear apparently a higher pathogenic potential for humans [27].

There are also smaller evolutionary intermediate isolate groups, which are for example positive for *dmsA*, *ansB*, *cj1365c* and *fucP* but not for *ggt*[18,19].

Furthermore, MLST-ST 21 isolates have a variation of TLP7, which is expressed as dimer [18,41]. In this group of isolates the most *in vitro* hyperinvasive strains can be found [42]. These isolates are mostly responsible for outbreaks associated with cattle [17].

We have shown in this study that biomarker shifts can be used to discriminate not only between the vast majority of *C. jejuni* isolates and this *C. jejuni* subgroup with an extended amino acid metabolism (*ggt*^+^), which was shown to have a higher pathogenic potential for humans [27], we were even able to discriminate between MLST-CC 45/282 isolates and MLST-CC 22/42 isolates. MLST-CC 22/42 isolates positive for the LOS-sialyltransferase *cstII* could be associated with GBS and higher host cell invasiveness [19].

Furthermore, we were able to identify another biomarker ion (m/z = 5303) that differentiates the subset of MLST ST 21 isolates associated with the dimeric TLP7_m+c_-variant.

It should be noted that the biomarker ions are not based on the expression of the marker genes used, as the proteins encoded in the marker genes are of entirely different sizes than the observed masses, but there is an obvious evolutionary association between the presence of specific marker genes and some of the biomarker ions.

## Conclusions

In conclusion, our study demonstrates that it is possible to discriminate specific subtypes within the *C. jejuni* species that have a different metabolism and different clinical relevance even using smear spectra.

Phyloproteomics corresponds only partial to phylogenetics. However, the phyloproteomic relatedness reflects phenotypic aspects better than the phylogenetic and it therefore may present a more meaningful typing approach than MLST.

Nevertheless, before such subtyping approaches for use in epidemiology can be implemented in the respective commercial ICMS MALDI-TOF MS technologies using for example weighted pattern matching and specific reference spectra, additional approaches to increase the robustness of spectrum generation and clustering are necessary.

## Methods

### *C. jejuni* strains

For our analyses we chose a total of 104 *C. jejuni* isolates. Eventually, 46 isolates of human, 31 of chicken, 16 of bovine, and 11 of turkey origin, which had previously been characterized for 16 different genetic markers (the genes for: the serine protease *cj1365c*, the oxidoreductase *cj1585c*, the dimeric formic acid chemotaxis receptor *tlp7*_*m+c*_[43], the tripartite anaerobic dimethyl sulfoxide oxidoreductase subunit A *dmsA*, the periplasmic asparaginase *ansB*, periplasmic gamma-glutamyl-transpeptidase *ggt*, the *O*-glycosylation cluster *cj1321-6*, the fucose permease *fucP*, the outer membrane siderophore receptor *cj0178*, the iron uptake protein *cj0755*/ferric receptor *cfrA*, enterochelin E *ceuE*, phospholipase A *pldA*, lipooligosaccharide sialyltransferase II *cstII*, lipooligosaccharide sialyltransferase III *cstIII*, *Campylobacter* invasion antigen B *ciaB*, and cytolethal distending toxin subunit B *cdtB*) [18,19] were selected. The isolates were chosen in such a way that particular representative groups of MLST-related isolates with almost identical marker gene profile could be arranged (see Additional file 2: Table S2) and a wide spectrum of different MLST ST/CC was covered. Thus, three to five isolates with same or close related MLST CC(ST): 21(21, 50, 53), 206(46, 122, 572), 48(38, 48), 446(450), 49(49), 283(267), 45(45), 42(42), 828(828), 52, 443, 22(22), 353(353), 354(354), (464), 658(658), 61(68, 61), (877), 257(257), 1034 and a typical marker gene profile were selected. Isolates with an atypical marker gene profile and redundant isolates (with reference to the previous studies [18,19]) were not included.

Avian and bovine isolates were originally obtained from the German *Campylobacter* reference center at the *Bundesinstitut für Risikobewertung* (Federal Institute for Risk Assessment) in Berlin, Germany. The bovine isolates originated from anal swabs taken in 2004-2009, the turkey isolates from cloacal swabs taken in 2007-2009, and the chicken isolates from cloacal swabs taken in 2003-2009. All distributed over the whole area of the German federal republic. The human isolates originated from stool samples of patients with watery diarrhea (85%) or bloody diarrhea (15%) processed at the University Medical Center Göttingen, Germany in the years 2000 – 2004 [18,19].

### Culture conditions and intact cell mass spectroscopy

All isolates were grown in one batch under identical conditions on Columbia agar base (Merck, Darmstadt, Germany) supplemented with 5% sheep blood (BA) and incubated at 42°C under microaerophilic conditions (5% O_2_, 10% CO_2_, 85% N_2_) over night, prepared in duplicate for ICMS by smear preparation and overlaid with HCCA matrix. For reproducibility it was important to use exactly the same culture conditions (identical lot number of agar plates and identical size of anaerobic/microaerophilic culture jars) and to grow all isolates parallel in one occasion. Using the extraction method (harvesting and washing the cells in 70% ethanol, subsequent drying, and lysing the cells in 70% formic acid followed by ACN addition) demonstrates no significant differences in comparison to smear preparation.

ICMS was done by standard procedures recommended for the MALDI Biotyper system (Bruker Daltonics, Bremen, Germany). For analysis, 600 spectra from 2-20 kDa were gathered in 100-shots steps and added. Results with MALDI Biotyper identification score values ≥2.000 were considered correct. Analyses not yielding a significant score did not occur.

### PCA-analysis

Phyloproteomic analyses were done using Flexanalysis and the PCA-algorithms implemented into the MALDI Biotyper 3.0 software (both Bruker Daltonics, Bremen, Germany). Spectra were pre-processed by baseline subtraction and smoothing, for ICMS-spectra-based PCA hierarchical clustering distance measurement was set to ‘correlation’; the linkage algorithm to ‘average’. Recording of spectra and subsequent phyloproteomic analyses using the PCA-algorithms was performed four times, two times each using smear preparation and the extraction method. Before comparison of the obtained PCA-trees of all four biologically independent repeats the existing degrees of freedom were assessed and the dendrogramms were converted by pivoting single (sub-)branches around existing dendrogram nodes in such a way that phyloproteomic relatedness was visualized optimally.

### Phylogenetic analysis

For construction of a UPGMA-dendrogram (unweighted-pair group method using average linkages) the MEGA5.1 software was used [44], and the *C. jejuni* MLST website (http://pubmlst.org/campylobacter/) was consulted for designation of sequence types and clonal complexes [45].

## Abbreviations

MLST: Multilocus sequence typing; CC: Clonal complex; MS: Mass spectrometry; ICMS: Intact cell mass spectrometry; MALDI-TOF: Matrix-assisted laser desorption/ionization – time of flight; cj: gene numbering based on the genome sequence of *Campylobacter jejuni* strain NCTC 11168; GBS: Guillain-Barré-syndrome; tlp7m+c: transducer like protein 7 gene encoding the membrane associated part (*cj0952c*) and the cytoplasmic part (*cj0951c*) in two separate genes; dmsA: the tripartite anaerobic dimethyl sulfoxide oxidoreductase subunit A gene; ansB: periplasmic asparaginase gene; ggt: periplasmic gamma-glutamyl-transpeptidase gene; fucP: fucose permease gene; cfrA: iron uptake protein *cj0755*/ferric receptor; ceuE: enterochelin E gene; pldA: phospholipase A gene; cstII: lipooligosaccharide sialyltransferase II gene; cstIII: lipooligosaccharide sialyltransferase III gene; ciaB: *Campylobacter* invasion antigen B gene; cdtB: cytolethal distending toxin subunit B gene; HCCA: alpha-Cyano-4-hydroxycinnamic acid, ACN, acetonitrile.

## Competing interests

The authors declare that they have no competing interest.

## Authors’ contributions

Conceived and designed the experiments: AEZ OB UG. Performed the experiments: AEZ AMT WOM OB. Analyzed the data: AEZ OB. Contributed reagents/materials/analysis tools: AMT MW RL. Wrote the paper: AEZ OB WOM UG. All authors read and approved the final manuscript.

## Supplementary Material

Additional file 1: Table S1 Marker gene profile of 104 *C. jejuni* isolates given in the order of the ICMS-based PCA-dendrogram. Presence of a given marker gene is indicated in orange, absence is indicated in green. The group assignment in the last column is taken from a previous study [18]. Click here for file

Additional file 2: Table S2 Marker gene profile of 104 *C. jejuni* isolates given in the order of the MLST-based UPGMA-tree. Presence of a given marker gene presence is indicated in orange, absence is indicated in green. The group assignment in the last column is taken from a previous study [18]. Click here for file
