# Supplementary material for: Discrimination of multilocus sequence typing-based Campylobacter jejuni subgroups by MALDI-TOF mass spectrometry
Source: BMC Microbiol. 2013 Nov 7;13:247. doi: 10.1186/1471-2180-13-247 (PMC4228279; doi:10.1186/1471-2180-13-247)
Supplement: Additional file 1: Table S1 — Marker gene profile of 104 C. jejuni isolates given in the order of the ICMS-based PCA-dendrogram. Presence of a given marker gene is indicated in orange, absence is indicated in green. The group assignment in the last column is taken from a previous study [18]. [file 1471-2180-13-247-S1.pdf]

Supplementary Table 1: Marker gene profile of 104 *C. jejuni* isolates in order of the ICMS-based PCA-dendrogram

Isolate	origin	MLST-ST	MLST-CC	*cj1365c*	*cj1585c*	*dtlp7*	*dmsA*	*ansB*	*ggt*	*cj1321-6*	*Fucose*	*cj0178*	*cj0755*	*cstII*	*cstIII*	group
huA35	human	122	206													1B
HS01	chicken	877	none													5
huC22	human	584	257													6
huB11	human	257	257													6
BFR3895	human	45	45													2B
bovC537	cow	42	42													2A
huE23	human	257	257													6
huE15	human	4571	257													6
BFR3918	human	42	42													2A
bovC084	cow	4670	42													2A
av7107	chicken	42	42													2A
HW7	chicken	464	none													3A
av7270	chicken	2288	354													3A
HW4	chicken	464	none													3A
me680	turkey	464	none													3A
HW3	chicken	464	none													3A
meC0282	turkey	450	446													1B
HS8	chicken	4658	353													3A
huE2	human	49	49													1B
huA33	human	4590	49													1B
BFR3868	human	45	45													2B
BFR3951	chicken	46	206													1B
HW2	chicken	464	none													3A
huA34	human	572	206													1B
BFR3889	cow	61	61													3B
BFR3865	human	53	21													1A
BFR3876	human	53	21													1A
BFR3912	cow	61	61													3B
av7292	chicken	50	21													1B
bovC404	cow	21	21													1A
bovC567	cow	21	21													1A
bovC536	cow	21	21													1A
bov79	cow	21	21													1A
huE12	human	450	446													1B
HS9	chicken	353	353													3A
meC340	turkey	50	21													1B
av7126	chicken	353	353													3A
huA29	human	658	658													3A
huA13	human	3188	206													1B
av509	chicken	977	1034													4
huA4	human	4626	1034													4
BFR3947	human	60	61													3B
huC20	human	51	443													3A
HS16	chicken	4659	none													5
HS10	chicken	877	none													5
meC283	turkey	443	443													3A
bovC539	cow	38	48													1B
BFR3922	chicken	46	206													1B
meC816	turkey	1709	1034													4
av7297	chicken	50	21													1B
av7278	chicken	50	21													1B
huB12	human	257	257													6
huE11	human	4651	none													5
meC130	turkey	2288	354													3A
huA9	human	354	354													3A
huC25	human	775	52													3A
bov159	cow	572	206													1B
BFR3881	human	53	21													1A
huA23	human	267	283													2B
BFR3926	chicken	46	206													1B
huB6	human	4631	446													1B
huB16	human	354	354													3A
bov277	cow	352	61													3B
huA28	human	122	206													1B
HS5	chicken	1900	658													3A
huA26	human	572	206													1B
BFR3866	human	53	21													1A
huA6	human	4587	49													1B
HS7	chicken	353	353													3A
81-176	human	913	42													2A
BFR3943	human	60	61													3B
BFR3043	human	60	61													3B
av0097	chicken	122	206													1B
huB20	human	4637	52													3A
huC07	human	4643	658													3A
huE22	human	696	1332													4
BFR3907	human	267	283													2B
bovC246	cow	48	48													1B
meC0475	turkey	257	257													6
huC24	human	51	443													3A
huA22	human	4630	52													3A
av4116	chicken	450	446													1B
huB13	human	257	257													6
av7060	chicken	572	206													1B
BFR3885	cow	48	48													1B
av7073	chicken	48	48													1B
av7252	chicken	50	21													1B
BFR3948	chicken	49	49													1B
HS3	chicken	4656	353													3A
meC0280	turkey	828	828													3A
meC0281	turkey	828	828													3A
meC467	turkey	828	828													3A
bovC405	cow	38	48													1B
huC8	human	383	283													2B
BFR3950	chicken	564	283													2B
BFR3929	cow	45	45													2B
BFR3880	human	45	45													2B
BFR3867	human	45	45													2B
bovC117	cow	22	22													2A
BFR1764	human	22	22													2A
av5020	chicken	22	22													2A
huE19	human	22	22													2A
meC1496	turkey	4674	1034													4
av5018	chicken	22	22													2A
